# Two-Dimensional Radial Laser Scanning for Circular Marker Detection and External Mobile Robot Tracking

**DOI:** 10.3390/s121216482

**Published:** 2012-11-28

**Authors:** Mercè Teixidó, Tomàs Pallejà, Davinia Font, Marcel Tresanchez, Javier Moreno, Jordi Palacín

**Affiliations:** Department of Computer Science and Industrial Engineering, University of Lleida, Jaume II, 69, 25001 Lleida, Spain; E-Mails: mteixido@diei.udl.cat (M.T.); tpalleja@diei.udl.cat (T.P.); dfont@diei.udl.cat (D.F.); mtresanchez@diei.udl.cat (M.T.); jmoreno@diei.udl.cat (J.M.)

**Keywords:** laser scanning, laser, circle fitting, mobile robots localization

## Abstract

This paper presents the use of an external fixed two-dimensional laser scanner to detect cylindrical targets attached to moving devices, such as a mobile robot. This proposal is based on the detection of circular markers in the raw data provided by the laser scanner by applying an algorithm for outlier avoidance and a least-squares circular fitting. Some experiments have been developed to empirically validate the proposal with different cylindrical targets in order to estimate the location and tracking errors achieved, which are generally less than 20 mm in the area covered by the laser sensor. As a result of the validation experiments, several error maps have been obtained in order to give an estimate of the uncertainty of any location computed. This proposal has been validated with a medium-sized mobile robot with an attached cylindrical target (diameter 200 mm). The trajectory of the mobile robot was estimated with an average location error of less than 15 mm, and the real location error in each individual circular fitting was similar to the error estimated with the obtained error maps. The radial area covered in this validation experiment was up to 10 m, a value that depends on the radius of the cylindrical target and the radial density of the distance range points provided by the laser scanner but this area can be increased by combining the information of additional external laser scanners.

## 1. Introduction

The versatility and precision of the state-of-the-art two-dimensional laser scanners has enabled the development of a huge range of useful non-contact measurement applications. For example, Palleja *et al.*[1] proposed the use of a laser scanner to measure the parameters of the gait during straight displacement and Teixido *et al.*[2] proposed and enhanced an application to measure oscillating walking displacements which could have future applications in an early detection of different diseases that produce alterations in the gait, including osteoporosis [3], diabetes [4] or Parkinson’s [5,6]. A similar approach has been applied to forestry. For example, Miettinen *et al.*[7] and Zheng *et al.*[8] proposed the use of a laser scanner to locate trees, Lin *et al.*[9] to estimate the height of trees, and Jutila *et al.*[10] to estimate the diameter of trees. The information obtained with a laser scanner has been also used for indirect estimate of other forestry parameters. Palacin *et al.*[11] proposed indirect estimate of foliage surface and Palleja *et al.*[12] evidenced that small errors in the assumed spatial trajectory of the laser have strong influence in the parameters deduced.

In another range of applications, the use of laser scanners simplifies the location and tracking of mobile robots. Vázquez-Martín *et al.*[13] proposed the segmentation of the laser scan data to extract invariant features of the environment. Espinosa *et al.*[14] combined the internal robot odometry with the laser scan data. Zhang *et al.*[15] combined the information obtained from a laser rangefinder with the information extracted from a monocular camera to remove the information corresponding to moving objects in the description of the environment. All the cited papers focused the efforts on the mobile robot self-location problem, but there are many alternative applications that also require external verification or validation of the mobile robot trajectory [16], for example by using different odor sensors [17] to estimate gas or odor source concentration [18–21].

The new contributions of this paper are the proposal to use a fixed external laser scanner to locate and track the displacement of a circular marker on a two-dimensional plane and the determination of the distribution of the location errors originated in this. This proposal can be applied to externally track the trajectory of a mobile robot just by attaching a cylindrical target to its structure but also to track any moving device that will require external validation of its position or trajectory. This application can be enhanced in the future with the combination of the scan data obtained with different fixed laser scanners placed strategically in a given environment to increase the area covered by the measurement system. An additional advantage related to the use of a cylindrical target is that its circular perimeter description in the scan data is robust to large changes in height originated by irregularities in the terrain, thus enabling the development of trajectory tracking measurements in outdoor applications.

The rest of the paper is organized as follows: Section 2 describes the materials and methods required to detect circular markers; Section 3 describes the experiments carried out to measure the location error of different circular markers; Section 4 describes the validation experiment performed to locate and track a mobile robot; finally, Section 5 presents the conclusions of the paper.

## 2. Materials and Methods

The materials used in this paper are the UTM-30LX laser sensor manufactured by Hokuyo (Osaka, Japan), a rotating structure for the laser sensor which includes additional cooling, and cylindrical targets of different diameters for experimentation. The method used in this paper is the procedure applied to detect a circle in the raw scan data provided by the sensor.

### 2.1. Hokuyo UTM-30LX

**Figure 1.**
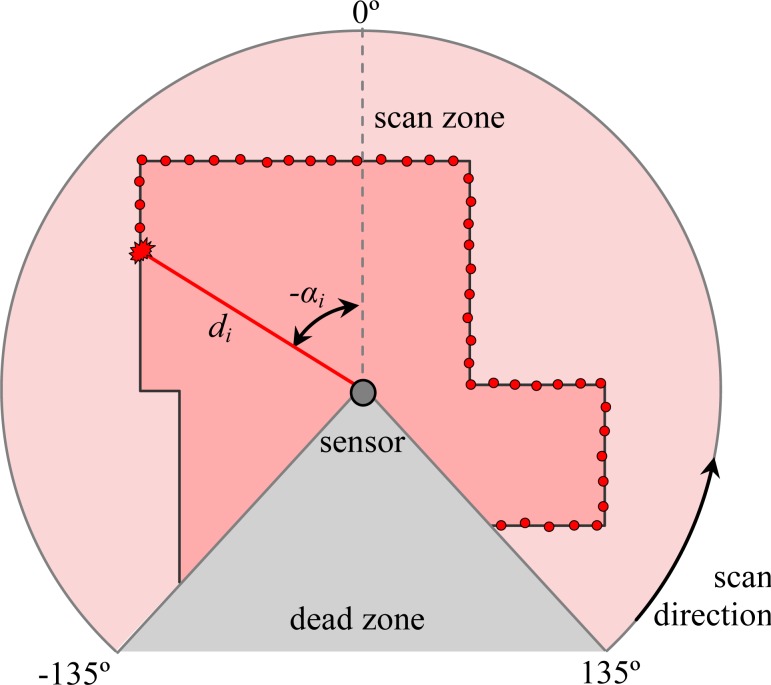
Sensor scanning.

The Hokuyo UTM-30LX [22] is a two-dimensional radial laser rangefinder that measures 1,081 distance points in a range from −135° to 135° where orientation 0° corresponds to the front of the sensor (see Figure 1), taking 0.025 s per scan when operating in the continuous acquisition mode. The laser scanner has a resolution of 1 mm in a range from 0.1 to 60 m and requires a dedicated power source (12 V, 1 A) to operate properly. In this paper, the result of the *i* measurement within a scan is represented in polar coordinates (*d_i_*, *α_i_*) where *d* is the distance measured and *α* the relative angle of the measurement. Figure 1 shows a simulated example of a sensor scanning a room: the small red circles represent the sequence of distances measured in the scan direction (from right to left), the dark area represents the dead zone behind the sensor where no information is available.

### 2.2. Rotating and Cooling Measurement Structure

**Figure 2.**
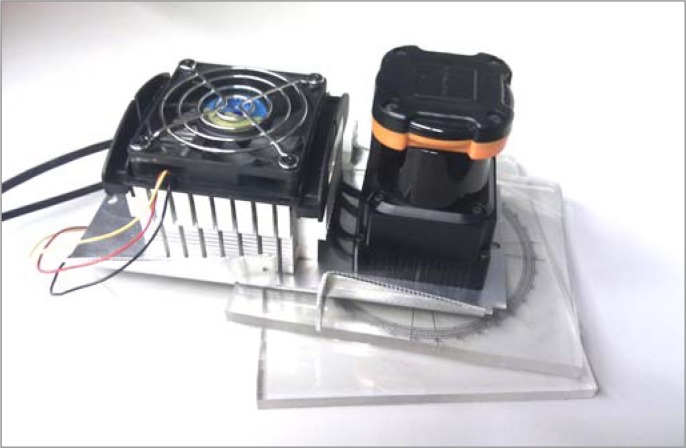
Laser sensor and additional cooler on a rotating structure.

Figure 2 shows the rotating and cooling measurement structure created for the experimental part of this paper. The manufacturer recommends the use of an aluminum plate (200 × 200 × 2 mm) as a heat sink structure [23] for the device to avoid temperature dependences. After a trial and error procedure, an additional PC cooler (see Figure 2) with a fan was added to the aluminum plate to improve the cooling of the device.

**Figure 3.**
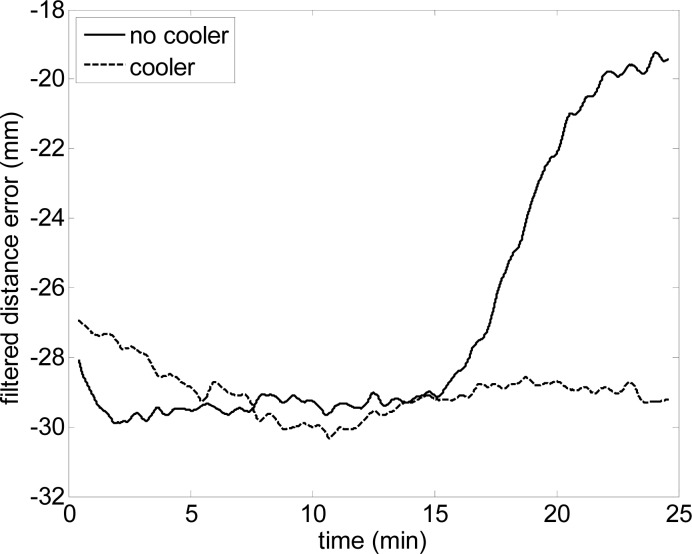
Dynamic evolution of the error in one distance measured with and without cooler.

Figure 3 shows the influence of the cooler on the distance measured by the laser sensor at *α = 0*° when a plain target was at *d* = 500 mm and perpendicular to the laser beam. The data corresponds to 60,000 scans acquired in approximately 25 min of continuous acquisition. The data shows the dynamic evolution of the relative error obtained in the distance measured by the sensor with and without the cooler. The data is filtered with a mean filter window covering 25 s of data samples to remove the measurement noise and show the temperature dependence. Figure 3 shows that the error induced by the temperature of the sensor increased after 15 min of use, reaching a maximum divergence of 10 mm between both cases at the end of the experiment. This error source can probably be neglected when the laser scanner is attached to a large mechanical structure that operates as a heat sink, but this is not the case of this paper as the planned support for the laser scanner was a rotating methacrylate structure whose thermal conductivity is 1,500 times lower than aluminum.

**Figure 4.**
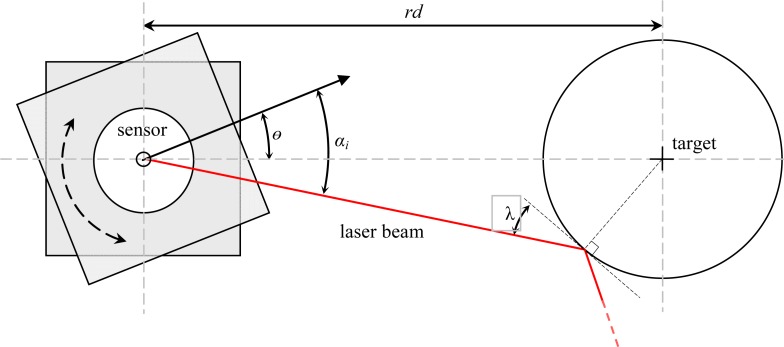
Schematic image of the rotating structure that holds the sensor.

The measurement structure has also two transparent methacrylate planes (see Figure 2) joined with a rotation axis to modify the relative orientation of the sensor easily (angle *θ* in Figure 4). One of the transparent methacrylate planes includes a printed angular reference to simplify visual adjustment of the orientation. A special effort was made to align the laser sensor’s center of measurement with the center of rotation of the methacrylate structure.

Finally, Figure 4 shows the representation of the real distance from the center of the laser sensor to the circular targets, *rd*, and the angle of incidence of the laser beam over the surface of the circular target, λ (with a theoretical range from 0 to 90°).

### 2.3. Cylindrical Targets

**Figure 5.**
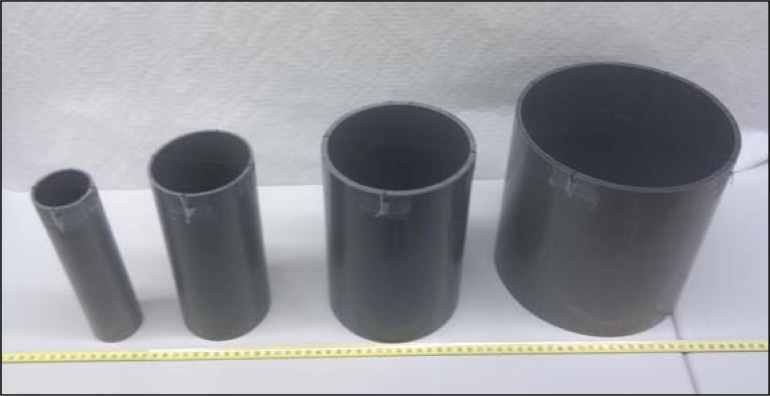
Set of PVC cylinders used as targets.

The cylindrical targets used in this paper were a set of standard low-cost PVC tubes (Figure 5) widely used in construction. The cylinders selected had a diameter of 50, 90, 140 and 200 mm and a height of 200 mm, but this value can be reduced to 100 mm or less depending on the expected elevation changes affecting the measurements. These empty cylinders can be attached to a mobile robot or any other moving device to externally estimate its position and trajectory; in all cases the proposed measurement system will register the location and trajectory of the center of the cylindrical target. It is strongly recommended to align the center of the target with the center of rotation or origin of references of the mobile robot or moving device registered to avoid additional trajectory conversions.

### 2.4. Method for Circle Detection in the Scan Data

There are many alternative methods for fitting circles to perimeter data points obtained with a laser scanner. Umbach *et al.*[24] reviewed five alternatives based on the Least Squares Method (LSM) [25] and analyzed their sensitivity to measurement errors and Chernov *et al.*[26] presented an extensive review of some methods for fitting circles based on the Levenberg-Marquardt algorithm (LMA) [27], but there are many other alternative methods. As an application example, Zheng *et al.*[8] compared different function minimization strategies such as the Fletcher and Reeves (F-R) [28] and a LMS to detect tree trunks in the data obtained with a laser rangefinder. The accuracy of the results with the different approaches was similar, but the F-R was 67% faster. Huang *et al*. [29] proposed the use of the Random Sample Consensus (RANSAC) iterative method for circle detection and fitting in the raw data obtained from a laser rangefinder, obtaining positioning errors of around 15 mm. This method consists of randomly choosing a subset of data which can determine a set of parameters of the model. This model is considered acceptable if there are enough points in the data set. Further specific information can be obtained in the detailed error analysis of the most popular methods for circle fitting performed by Al-Sharadqah *et al.*[30].

The method used for circle fitting in this paper was the LSM with the LMA that provides a numerical solution to the problem of minimizing a function over a space of parameters of the function. The LMA only finds a local minimum but in this paper the space of parameters of the circular fitting is limited because the radius of the circle is known and a rough estimate of the coordinates of the centre of the marker is also available. Therefore, the local minimum found in this space of parameters is always very close to the global minimum of the function.

**Figure 6.**
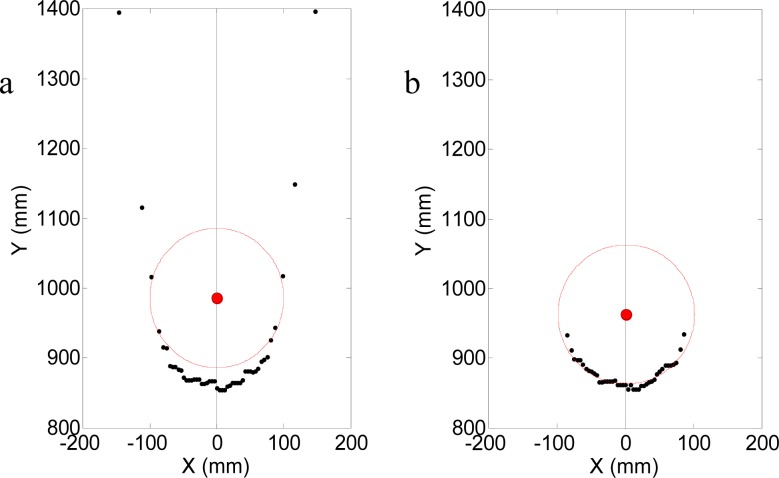
Sample circle fitting with (**a**) and without outliers (**b**).

Equation (1) shows the objective function to minimize, *F*:

(1) $$F=\sum_{i=1}^{n}\sqrt{{\left(x_{i}-xc\right)}^{2}+{\left(y_{i}-yc\right)}^{2}}-R$$

where (*x_i_*, *y_i_*) are the Cartesian coordinates of a subset of the available scan points that define the circle of the target, *R* is the known radius of the target, and (*xc*, *yc*) are the unknown Cartesian coordinates of the center of the circle. Figure 6 shows two examples of results from a circular LSM fitting with distance points obtained with the laser system. In the case of Figure 6(a), the subset of the fitting points includes some outlier points, whereas in Figure 6(b), the circle outliers have been removed from the fitting points. In this sample case, the presence of outliers generated an error of 14 mm in the estimate of the center of the cylindrical target, so special attention must be given to removing the outliers from the subset of scan points used in the fitting process.

**Figure 7.**
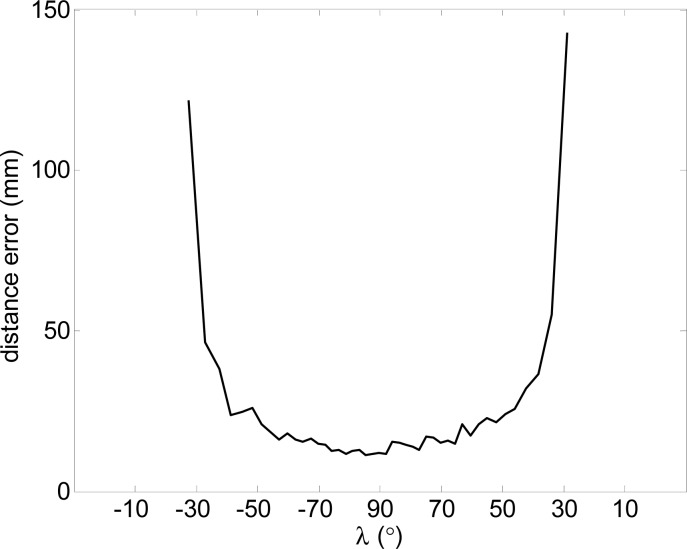
Maximum error in individual distance measurement relative to the angle of incidence λ.

In general, the outliers appear for small angle of incidence λ (see Figure 4) and correspond to circular boundary points. To illustrate this description, Figure 7 shows the maximum error obtained in the measurement of one distance when changing the angle of incidence, λ, (90° is when the laser is perpendicular to the target), the error is very high when |λ| is lower than 30° and can have very large values depending on the distance from the target to the existing background (see Figure 6).

The following two steps method is proposed to avoid the outliers from the fitting data: (1) performing a first rough estimate of the target center from the subset data points with:

(2) $$\{\begin{array}{l} \omega =\text{min}(\alpha_{i})+\frac{1}{n}\sum_{i=1}^{n}\alpha_{i}\\ h=\text{min}(d_{i})+R \end{array}$$

where *n* is the number of points initially selected for fitting and (*h*, *ω*), the estimate of the distance and angular orientation of the center of the circle expressed in polar coordinates that can later be converted into Cartesian coordinates as (*x̂c*, *ŷc*). Taking advantage of the known radius *R* of the target and (2) computing the distance from the subset points to the estimate of the center, discarding as outliers all scan points with a distance higher than a threshold value that has been estimated as 1.5·*R* after analyzing the histogram of the distances obtained for the external circular data points.

## 3. Experimental Section

The experimental part of this paper was focused on analyzing the error obtained in the estimate of the position of the center of the cylindrical targets proposed. All experiments were performed in indoor facilities with an average illumination intensity of 520 lux, temperature of 26 °C, and humidity of 46%.

### 3.1. Evaluation of the Positioning Error Relative to the Distance for θ = 0°

**Figure 8.**
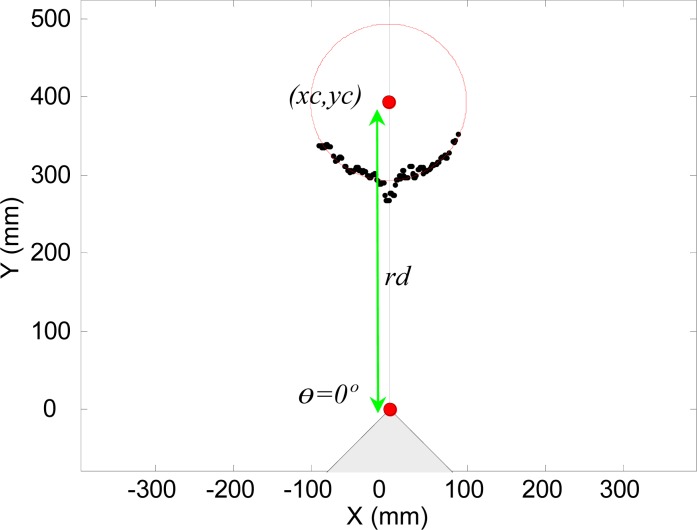
Example of circular target location.

**Figure 9.**
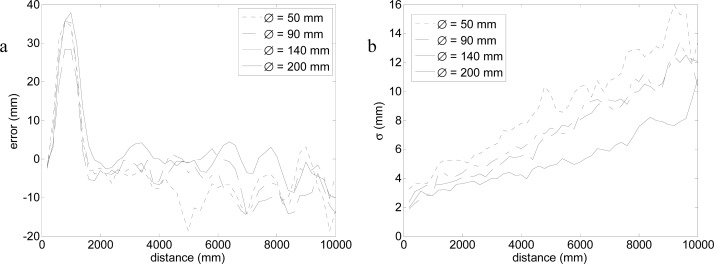
Average error (**a**) and standard deviation (**b**) for different target diameters.

The first experiment was prepared to evaluate the error in the measurement of the center of a circular target relative to the distance from the laser sensor for a fixed frontal sensor orientation (*θ* = 0°). This error was evaluated by placing the center of the different circular targets available at distances, *rd*, from 200 to 10,000 mm in increments of 200 mm, and acquiring 500 scans in each position. Figure 8 represents an example that shows the relative position of a target and the fitting points selected to estimate the center of the target in one particular case and Figure 9 shows the average results obtained.

Figure 9 shows the average error and standard deviation obtained at different distances and for different target diameters. In all targets, the average error (Figure 9(a)) increased in the distance range from 200 to 1,000 mm, decreased in the distance range from 1,000 to 2,000 mm and were almost constant in the range from 2,000 to 10,000 mm. Currently, the underlying causes of this unexpected error peak at distance 1 m and orientation 0° are unknown. The standard deviation (Figure 9(b)) increased with distance, with a slope that was inversely proportional to the diameter of the target. That is, a big circular target has more laser impacts (fitting points) and its center can be estimated more precisely.

### 3.2. Evaluation of the Positioning Error Relative to the Orientation for rd = 1,000 mm

**Figure 10.**
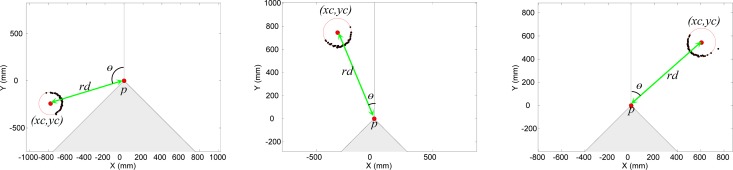
Circular target location for three different orientations.

The second experiment was designed to evaluate the error in the measurement of the center of a circular target relative to the orientation of the laser sensor at a fixed sensor distance (*rd* = 1,000 mm). This error was evaluated by placing the center of the different circular targets available at angles, *θ*, from −135 to 135° in increments of 5°, and acquiring 500 scans in each position. Figure 10 represents three examples showing the relative position of a target and the fitting points selected to estimate the center of the target in each particular case.

**Figure 11.**
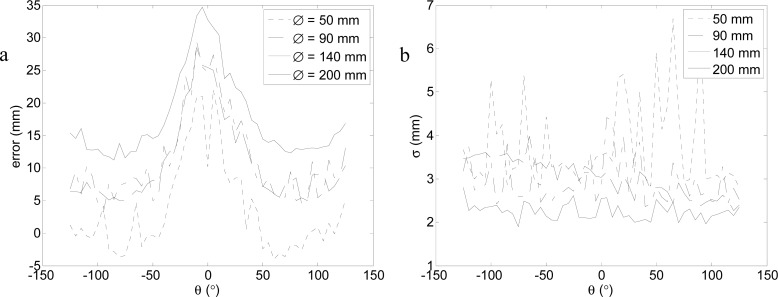
Average error (**a**) and standard deviation (**b**) for different target diameters.

Figure 11 shows the average error and standard deviation obtained at different orientations and target diameters. In all targets, the average error (Figure 11(a)) was higher when the target was in front of the sensor (*θ* ≈ 0°), but also lower for large-diameter targets. The standard deviation (Figure 11(b)) was almost plain (except in the diameter 50 mm case) and also decreased as the target diameter increased. There is a small discrepancy in the average error peak values shown in Figures 9(a) and 11(a) that are caused by the erratic non-Gaussian instantaneous error peak value originated at this distance for orientation 0°.

**Figure 12.**
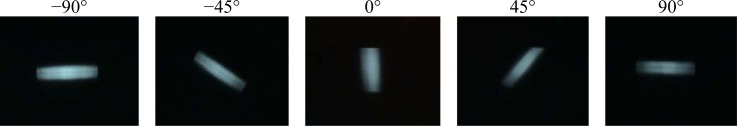
Shape of the laser beam at different scanning angles.

The justification for the angular orientation dependence of the results (Figure 11(a)) can be found in the rotation applied to the rectangular laser beam to perform the two-dimensional scanning. This effect is well known and has been reported in many two-dimensional laser scanners [31]. Figure 12 shows the rectangular shape of the laser beam acquired at different scanning angles, *α*. At 0°, the laser beam was completely vertical whereas at |90°|, it was horizontal. These images were obtained with a Sony^®^ Handycam DCR-SR72E digital video camera recorder operating in the 1-Mpixel photo mode. The camera and laser were in a dark room at a distance of 1,000 mm.

### 3.3. Evaluation of the Positioning Error for Different Distances and Orientations

The third experiment was a general evaluation of the error obtained in measuring the center of a circular target relative to the distance, r*d*, and orientation, *θ*, of the laser sensor. In this experiment, each cylinder (diameters: 50, 90, 140 and 200 mm) was placed at distances of 1,000 mm and from 2,000 to 10,000 mm in steps of 2,000 mm, and with orientations from −120° to 120° in steps of 20°. A total of 500 scans were acquired in each position. The circular fitting was performed if the surface of the cylinder was defined by at least 3 distance points; this value limits the distance range between the laser and the circular markers to 3,819, 6,875, 10,695 mm for marker diameter 50, 90, 140 and 200 mm, respectively.

**Figure 13.**
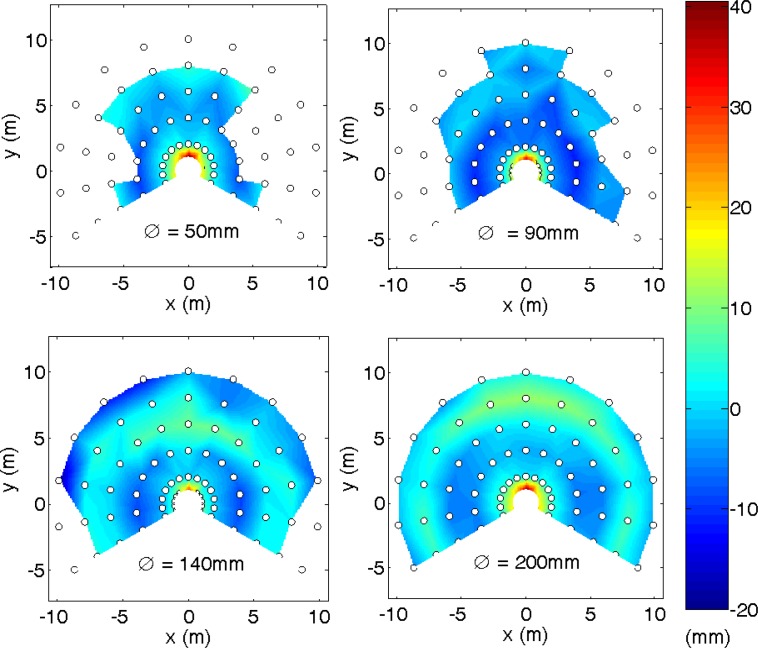
Average distance error map expressed in mm relative to the target.

**Figure 14.**
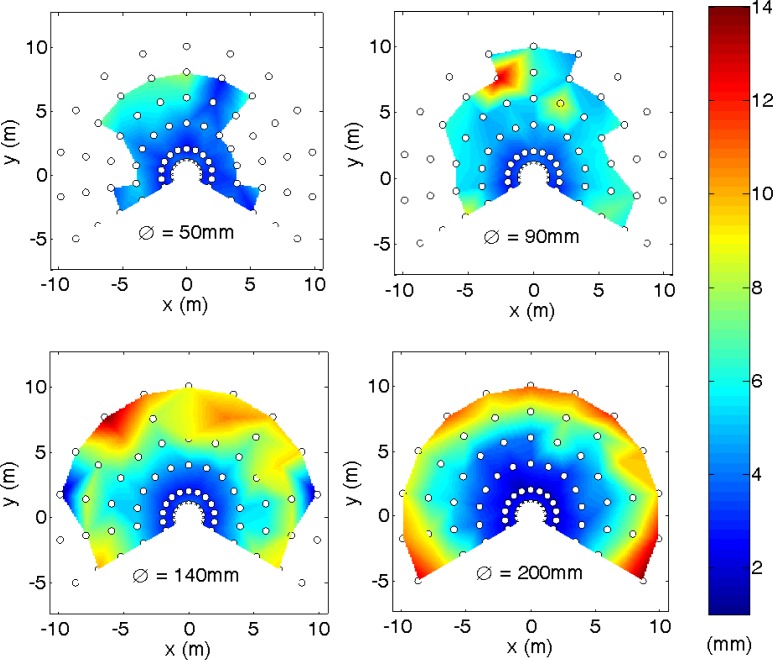
Standard deviation error map expressed in mm relative to the target diameter.

Figures 13 and 14 summarize the results obtained represented in a two-dimensional interpolated color representation. The white circles depict the measurement points and the intermediate data that was interpolated by the Triangle-based linear interpolation algorithm, also based on a Delaunay triangulation, which uses the Quickhull algorithm [32].

Figure 13 shows the average target location for the four diameters considered. It shows that the range of utilization increased with the diameter of the target and that the average error was also lower because more fitting points were available in the LSM adjust. The peak observed previously in Figures 9 and 11 was clearly visible at very short distances and orientations from −25° to 25°. Figure 14 shows the average target location for the four diameters considered. In general, the standard deviation increased as the distance of the circular target also increased. Therefore, given a target location obtained with circular fitting, the color map distribution shown in Figures 12 and 13 can be used to estimate the average positioning error and standard deviation of the localization performed.

## 4. Mobile Robot Tracking

**Figure 15.**
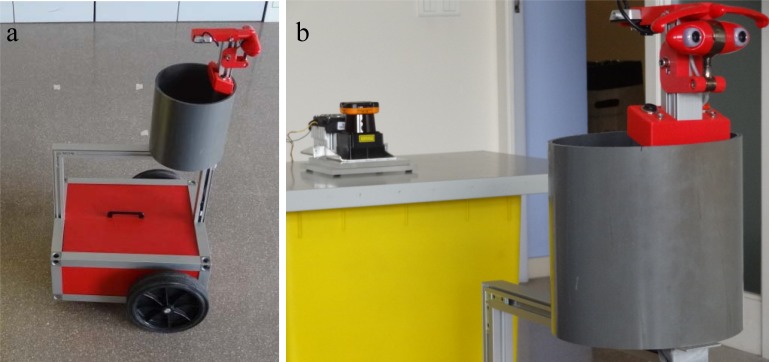
Images of the mobile robot with a circular marker attached (**a**) and the laser scanner (**b**).

The goal of this section was to validate the proposed measurement methodology by performing a mobile robot tracking experiment. To this end, a 200 mm diameter cylindrical target was been attached to a medium-sized general-purpose mobile robot called rBot, with the axis of the cylinder aligned with the axis of rotation of the mobile robot. Figure 15 shows two images of the mobile robot with the cylindrical target and the laser scanner during this tracking experiment. The measurement area was approximately 3 × 4.5 m and the measurement plane of the laser scanner was placed half way up the cylindrical target to assure robustness against ground irregularities.

**Figure 16.**
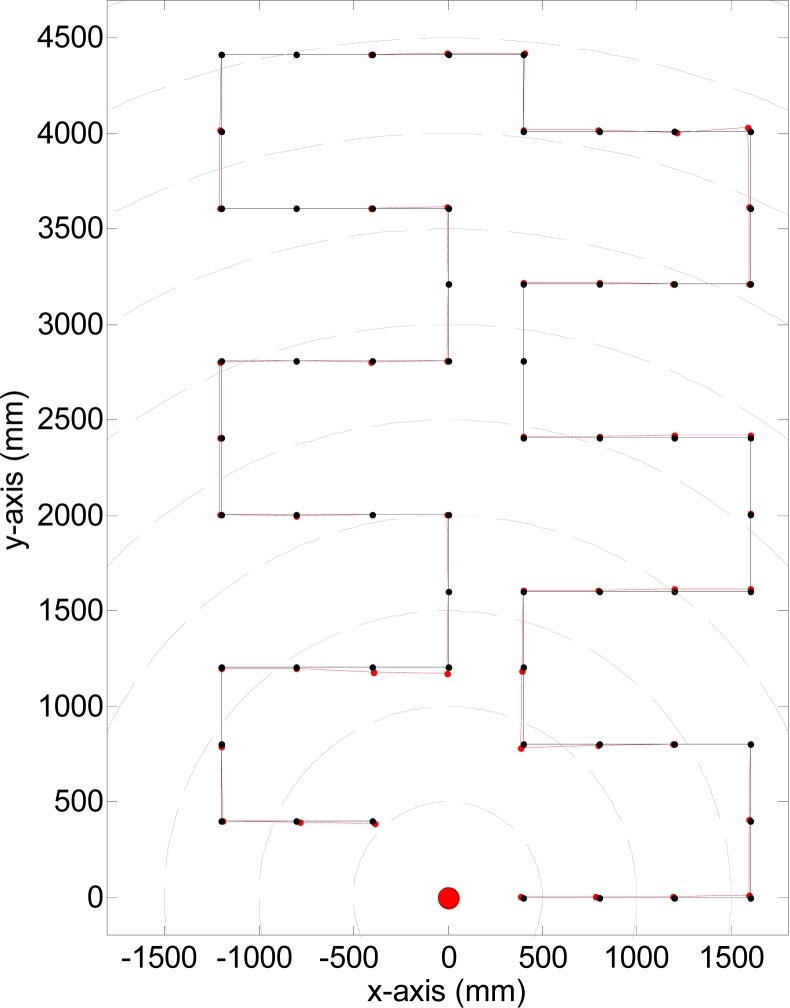
Comparison between the real and measured robot trajectory.

The black line in Figure 16 shows the trajectory followed by rBot in this experiment and the dots represent the measurement points achieved. This explorative trajectory was bio-inspired but was simulated, that is, the mobile robot was moved manually and its center of rotation placed in selected intermediate positions. The mobile robot includes additional reference marks to simplify its manual placement in the ground according selected intermediate trajectory positions with an error lower than 1 mm. This position was then measured with the laser measurement system and compared with the known absolute position. The red line in Figure 16 represents the measured trajectory and the red dots, the measurement points. Both trajectories, real and measured, are almost indistinguishable because of the large scale of the measurement area.

**Figure 17.**
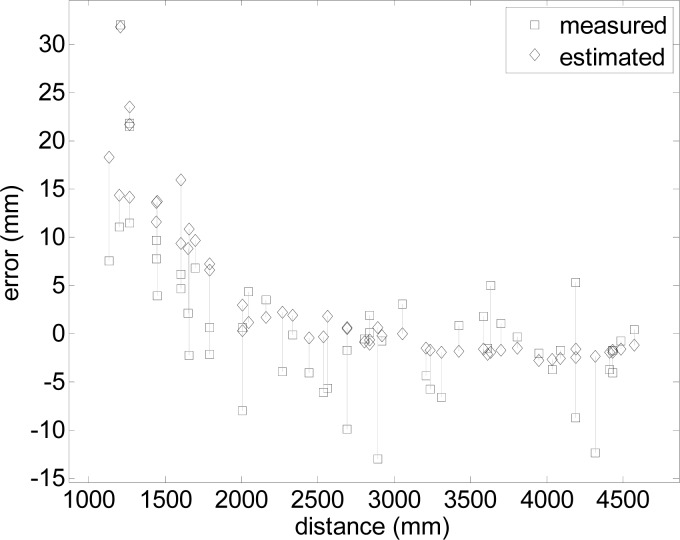
Placement error for different radial target distances.

The square marks in Figure 17 show details of the absolute error in the measurement of the center of the cylindrical target. As expected, the worst error was found at short distances (between 1,000 and 2,000 mm) with an error lower than 15 mm at longer distances. Additionally, the diamond marks in Figure 17 show the absolute error estimated from the data-maps shown in Figure 13 where a vertical line links the real error of the measurement and the average estimated error obtained from the data-maps in a given position. The agreement between both errors, measured and estimated, is very good, with an average divergence of 4.28 mm. Therefore, the conclusion of this experiment with a two-dimensional radial laser scanner is that the position of a circular marker attached to a mobile robot can be estimated with an error of less than 15 mm, while the measurement error maps deduced in this paper can be used to estimate the average error and standard deviation expected in any estimated position.

## 5. Conclusions

This paper proposes the utilization of a two-dimensional radial laser scanner to detect and locate cylindrical targets. This proposal has many applications, such as the external tracking of mobile robots. The laser sensor used in this paper was the UTM-30LX, which provides 1,081 range measurement points in a range from −135° to 135° and from 0.1 to 60 m. The targets used were based on standard PVC tubes. The laser scanner was equipped with an additional cooling structure to minimize possible temperature dependences during the measurement experiments. The procedure used for circle fitting from the scan data was based on the least squares method combined with an additional procedure to remove circle outliers.

Different experiments were developed to evaluate the positioning error of this location method. In a first experiment focused on determining the error in the estimate of the center of a circular marker at different distances and a fixed orientation, the conclusion was that the average error is lower than 20 mm, except at a distance of 1 m where the average error has a peak of 30–40 mm. In a second experiment focused on measuring the error in the estimate of the center of a circular marker at different orientation and a fixed distance (1 m), the conclusion was that the maximum average error at this distance is in a limited range in front of the sensor. In a third experiment, a general evaluation of the error in the estimate of the center of a circular marker for different distances and orientations was performed to obtain the two-dimensional distribution of the location error. The general conclusion was that the best target was the circular marker with the largest diameter.

Finally, a validation experiment with a mobile robot was performed to demonstrate the tracking capabilities of this proposal in a measurement area of 3 × 4.5 m. On average, the location error was less than 15 mm and this average value can be estimated by using the distribution maps also provided in this paper. This proposal will be used in the future to verify the exploratory trajectory followed by different mobile robots equipped with specialized sensors to detect gas plume sources and hazardous substances.
